# Are the Effects of Use and Disuse Inherited? An Examination of the View Held by Spencer and Darwin

**Published:** 1891-01

**Authors:** 


### REVIEW DEPARTMENT.

ARE THE EFFECTS OF USE AND DISUSE INHERITED? AN EXAMINATION OF THE VIEW HELD BY SPENCER AND DARWIN. By William Platt Ball. Macmillan & Co. London and New York. 1890.

Perhaps there is no feature in the theory of evolution more fascinating than that which concerns the transmissibility of individual characters, whether congenital or acquired. We love to consider that, to a great extent, the destiny of posterity is in our hands, and that as one generation shapes its conduct and its actions it will proportionately determine, in the same direction, the conduct and actions of many generations destined to live after it. We imagine to ourselves that we are the necessary product of those who preceded us 500 years ago, and that as our early progenitors lived and acted, as they kept their lives within the bounds of reason and moderation, or gave free rein to their passions, they impressed those traits of character on those who came after them, so that, by a species of reversion, corresponding vices or virtues cropped to the surface. But the author of the extremely suggestive volume we are reviewing rejects this pleasing doctrine so far as acquired traits are concerned. He admits, indeed, the plausibility of the doctrine and approaches its discussion with diffidence, as it has received the sanction which such names as those of Darwin and Spencer could not but assure even to a less tenable opinion. These scholars hold that acquired peculiarities impress themselves on the reproductive elements, and are thus transmitted to posterity. But, as our author well observes, if such transmissibility of acquired habits, modes of thought, tastes, proclivities, etc., were actually realized, it would be possible to modify the race profoundly and at once, and we would seek to improve its moral character by a judicious system of stirpiculture rather than by the inculcation of moral precepts. Much of the confusion that surrounds this question has arisen from the tendency to mistake congenital peculiarities, the result of natural and sexual selection, with peculiarities acquired during the lifetime of an individual. Actual experience and observation cannot aid us in the solution of the problem, since changes that are due in reality to general selection (natural, acquired or sexual), are equally dependent for their accomplishment on infinite lapses of time.

The author chiefly addresses himself to a refutation of the arguments employed by Herbert Spencer in support of his opinion as to the transmissibility of traits lost or acquired by use or disuse. The chief of these acquirements is that borrowed from the more powerful jaws of negroes and Australians, a peculiarity supposed to result from increased use employed by those savages in rending their coarse and unprepared food. But, our author remarks, why should this increased osteosity extend to the rest of the Tasmanian and African crania? Surely it is not that the occiput and sinciput of the savage has ever been brought into more active use. We must consequently look for another explanation of the lighter jaw of the civilized European, and it is most probable it may be found in natural selection. "Human preference, both sexual and social, would tend to eliminate huge jaws and ferocious teeth when these were no longer needed as weapons of war or organs of prehension." Besides, as against Mr. Spencer's position, it may be stated that the excessive heaviness of the jaw is not a uniform feature of the skulls of savages, as it should be were increased use an efficient cause of an abiding change. We have given this synopsis of Mr. Ball's arraignment of Herbert Spencer's doctrine in order to show in what manner he himself explains changes which the great apostle of evolution sets down to the operation of use and disuse. With Mr. Ball natural selection is all potent for the explanation of structural changes and the functional peculiarities that ensue. Thus, in regard to lap-dogs, artificial selection with a view to gentleness, would account for the enfeeblement of the whole biting apparatus without suggesting disuse as its cause. Perhaps the most frequently quoted instance of the influence of use is that of the giraffe. In the case of this strangely constructed animal it is not at all necessary to have recourse to the hypothesis of use-inheritance, since its phenomenal length of neck may be more naturally accounted for by the law of natural selection that enabled the tallest of the species to survive a period of famine and so transmit a selected peculiarity to its descendants. The author pursues the same line of argument in the chapter on "Æsthetic Faculties," and conclusively proves that here use-inheritance cannot be invoked as an explanation of the possession of this faculty in its higher forms. In respect to musical aptitude, it is remarkable that the appreciation of harmonious sounds is enkindled at a very early age in those who develop extraordinary musical tastes and capacities out of all proportion to ancestral acquisitions. Sexual selection is the most important factor that prevails in the development of musical tastes, since it is a well-known fact that persons possessing this æsthetic faculty are even more powerfully attracted toward each other than by beauty of face and figure.

What we have hitherto noticed in Mr. Ball's interesting monogram has reference to the supposed effects of use in developing racial peculiarities; and we have seen that he considers positive selection, both natural, sexual and artificial, as affording a suitable solution of the difficulties which the Spencer and the Lamarckian evolutionists have endeavored to explain by a reference to use-inheritance. But the negative results of disuse are equally valuable in supporting the position Mr. Ball has taken. Thus, in the case of the reduced wings of certain birds that inhabit oceanic islands, it is easy to account for this peculiarity without having recourse to the disuse theory.

It is not at all unlikely that in the course of a given migration a few birds of feebler wing structure barely succeeded in reaching those islands, and were compelled to stay there in consequence. Then the law of natural selection went into operation, and adaptation to environments began. In general, then, the elimination of useless organs is a consequence of that inexorable law of nature which continually tends to establish an equilibrium between organism and environment. Mr. Ball, in his chapter on the wings and legs of ducks and fowls, appeals to this law constantly, and proves against Darwin that the reduced wings and thickened legs of domestic fowl are the result of artificial and natural selection, under circumstances most favorable to the production of such results. It must likewise be borne in mind that in estimating the supposed consequences of disuse in individual specimens there is danger always of mistaking the acquired results of disuse for the transmitted consequences thereof, and thus confounding atrophy with disuse-inheritance. This very important distinction should be constantly borne in mind, and it seems to me that both Spencer and Darwin frequently overlooked it. Perhaps one of the most interesting chapters in Mr. Ball's work is that which concerns the true relation of parents and offspring. It is almost a popular belief that the physical, moral and intellectual peculiarities of progenitors are directly transmitted to those who spring from them, so that the saying, "as the parent is, so is the child," has almost passed into an aphorism. But the truth is a parent transmits not himself and his modifications, but the stock, the type, the representative elements of the species to which he belongs. Should a flaw exhibit itself in an individual, it is quite possible to find it reproduced in the offspring; but any general resemblance, as a result of acquired peculiarities in the parent, does not exist. Indeed, on the contrary, it does occur, and Galton has verified the fact that acquired excellences are often followed by corresponding defects in subsequent generations. The explanation of this interesting peculiarity of heredity is found in the fact that when the nervous system is developed by prolonged exercise, and there is a consequent improvement in the individual faculties, the best and most highly developed cells are used in the process, and the inferior ones are employed in reproduction. "Hence," says Mr. Galton, "the strong tendency to deterioration in the transmission of every exceptionally gifted race."

But our author goes further than to deny the existence of use-inheritance; he even points out that if it did exist it would work for decided harm. Thus those organs which in the human family are most constantly in use, would go on taking increase of growth from generation to generation till they would become unshapely and unwieldly. The heart, for instance, which is never at rest, would become unduly hypertrophied, and the molar teeth lengthened by extra use would not permit the incisors to meet. Here, again, it is well to bear in mind that the so-called effects of use and disuse are frequently the result of natural selection and the law of the survival of the fittest, for organisms become through use adapted to their surroundings, and we are prone to assign to the former what are the clear results of the operation of the latter. Lamarck, who gave so much prominence to the influence of use and disuse, in the theory with which is name is identified,

ARMY MEDICAL LIBRARY
WASHINGTON, D. C. U. S. A.

did not understand the law of natural selection as conceived by Darwin, so that we may not wonder at the errors into which he fell; but it is hard to understand how the philosopher of modern evolution should strive to perpetuate such mistakes. Mr. Ball's work is a timely and ingenious contribution to scientific progress, and will be read with interest even by those who may not accept his arguments or agree with his conclusions.

C. M. O'L.

